# Precursor B-cell lymphoblastic leukemia of the arm mimicking neurogenic tumor: case report

**DOI:** 10.1186/1477-7819-10-140

**Published:** 2012-07-10

**Authors:** Xiu-fang Sui, Wei-yong Liu, Wen Guo, Fang Xiao, Xing Yu

**Affiliations:** 1 Department of Ultrasound, The Anhui Province Hospital, Anhui Medical University, Hefei, 230001, China; 2 Department of Internal Medicine, Anhui Provincial Authority Hospital Communist Party of China, Hefei, 230001, China; 3 Department of Radiology, The Anhui Provincial Hospital, Anhui Medical University, Hefei, 230001, China; 4 Department of Emergency Medicine, The Anhui Provincial Hospital, Anhui Medical University, Hefei, 230001, China

**Keywords:** Precursor B-cell lymphoblastic leukemia, Arm mass

## Abstract

Precursor B-cell lymphoblastic lymphoma (PBLL) is an infrequent subtype of lymphoblastic lymphoma (LBL) that commonly affected site for the diagnosis is the skin, followed by the head and neck. In this report, we presented a special case of PBLL located at the left arm and detected with magnetic resonance imaging (MRI) and ultrasonography (US). This kind of PBLL is similar to a peripheral nerve tumor in clinical and radiographic manifestation.

## Background

It is already documented that lymphoblastic lymphoma (LBL) is an uncommon entity of non-Hodgkin’s lymphoma (NHL) with an incidence of less than 1% for B-cell LBL among all NHLs [1,2]. B-cell LBL is composed of immature lymphocytes that express precursor and B-cell markers. Although 80% of precursor B-cell neoplasms present as acute leukemia, a small proportion could manifest as a mass lesion [3]. We report here a 22-year-old man with an unusual precursor B-cell lymphoblastic lymphoma (PBLL) presenting as a mass in the left arm and extending along the neurovascular bundle, similar to a neurogenic tumor.

## Case presentation

A 22-year-old man was referred to a physician complaining of severe pain for 6 months from a progressively growing mass in the left arm. Medical history was unremarkable and the patient was not receiving any medication. He denied weight loss, night sweats and other unusual symptoms. Physical examination identified a fusiform palpable mass extending from the middle of the left arm to the elbow, combined with muscle weakness and numbness on forced flexion and supination. The laboratory results of kidney and liver functional tests, as well as the white blood cell counts and serum lactate dehydrogenase (LDH) levels, were within the normal range. The cerebrospinal fluid immunocytochemical analysis, bone marrow aspiration and biopsy showed negative for any malignancy. In addition, no positive symptoms were found on chest radiography or abdominal ultrasound (US).

**Figure 1:**
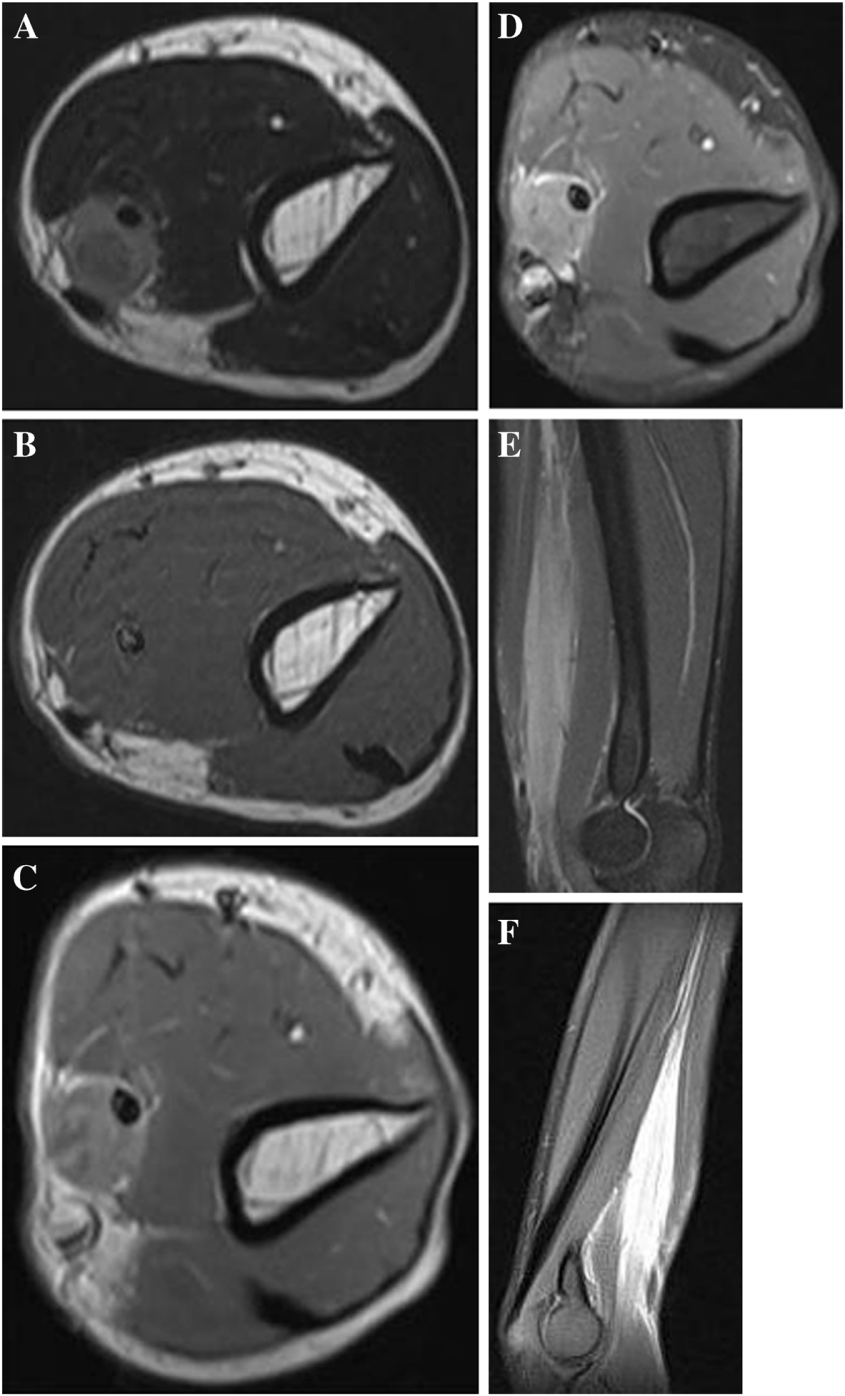
**The axial T2-weighted image revealed a homogeneously intermediate signal lesion located within the medial compartments in the left arm.** The hypo-intense median nerve is completely encased by the round-shaped mass (**A**). On the axial T1-weighted image (**B**) the lesion is isointense to skeletal muscle. The axial enhanced T1-weighted image (**C**) and axial fat-saturated enhanced T1-weighted image (**D**) show mildly uniform enhancement and a small area of necrosis. The coronal fat-suppression proton-density fast spin echo image. (**E**) demonstrates the mass in direct continuity with the median nerve with a fusiform shape. A well-defined lesion is noted on the coronal blade fat-suppression proton-density image (**F**).

Magnetic resonance imaging (MRI) revealed a well-defined mass located in the medial compartment of the left arm, with homogeneous and intermediate signal intensity on the axial T2-weighted image (Figure 1A) and isointense to skeletal muscle on the axial T1-weighted image (Figure 1B). The unusual thickened median nerve was completely encased by the lesion. The axial enhanced T1-weighted image (Figure 1C) and axial fat-saturated enhanced T1-weighted image (Figure 1D) mainly showed mainly uniform enhancement in accordance with the median nerve. The coronal fat-suppression proton-density fast spin echo image showed the mass longitudinally extended along the neurovascular bundle in extension to the median nerve with measures of 10 × 2 × 2 cm (Figure 1E). Well-defined margins were noticed on the coronal blade fat-suppression proton-density image (Figure 1F).

**Figure 2:**
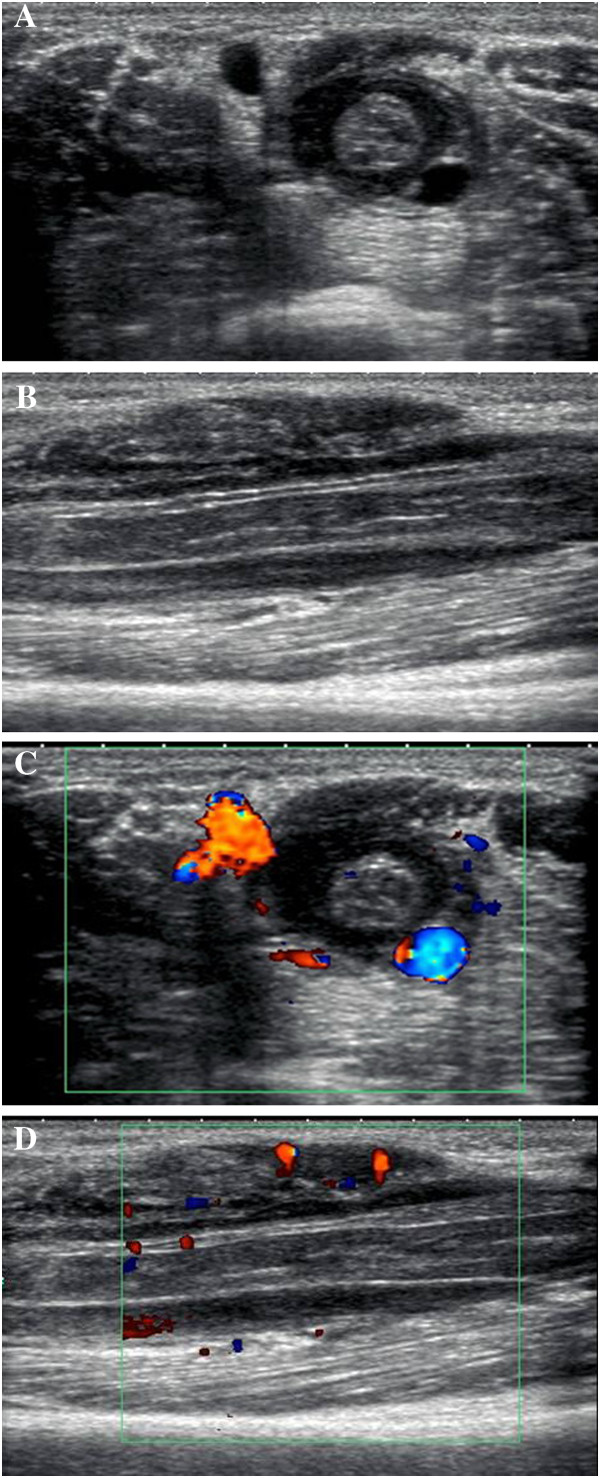
**Sonography images demonstrated a lesion encased the swollen median nerve.** (**A**) The transverse sonogram shows a round hypoechoic halo with well-defined margins and posterior acoustic enhancement completely surrounded the swollen median nerve. The brachial artery was partially encased, while the basilic vein and ulnar nerve were unaffected. (**B**) The longitudinal sonogram shows the mass extended along the median nerve. The swollen nerve shows hypoechoic echotexture lesion with discernible fascicles. The adjacent biceps brachii muscle with inhomogeneous-reduced echogenity is noted. (**C**) Avascularity could be visualized on the color Doppler image inside the mass. (**D**) Slightly increased vascularity is observed on color doppler sonography in biceps brachii muscle adjacent to the lesion.

High-resolution US established a marked hypoechoic lesion on the left distal arm. The hypoechoic lesion showed a clear round halo which was completely and concentrically encased in the median nerve in transverse sonograms. The median nerve was much larger than normal and was characterized by swollen fascicles (Figure 2A). Longitudinal sonograms revealed the well-defined lesion extending along the median nerve (Figure 2B). Color Doppler sonography demonstrated avascular flow signals in the lesion (Figure 2C). The margin of the biceps brachii muscle slightly abutting the mass showed some hyervascularization on the color Doppler image (Figure 2D), while the basilic vein and ulnar nerve were normal.

**Figure 3:**
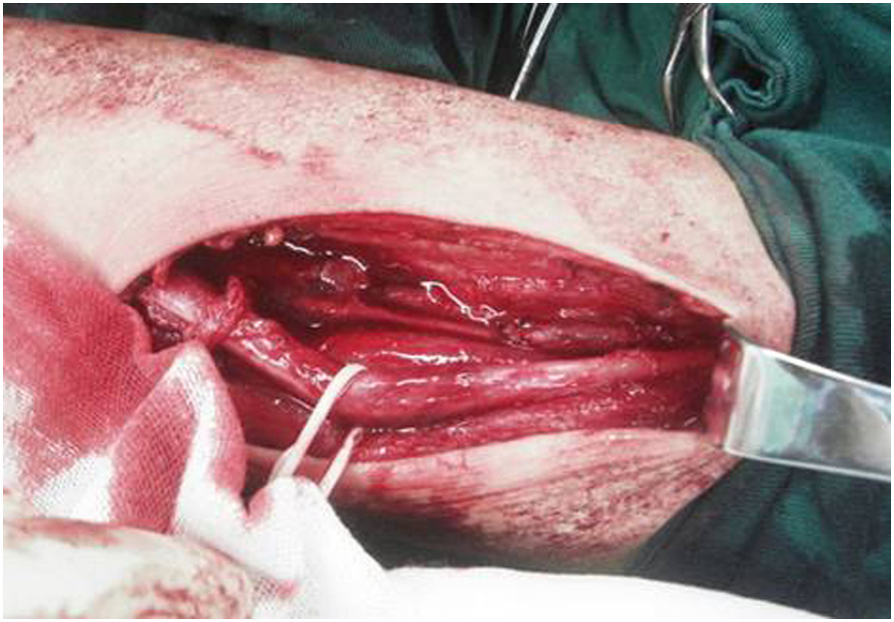
The intraoperative photograph shows the swollen and enlarged median nerve after resection of the lesion.

Through surgery, a well-defined and fusiform mass was found on the left arm. The tumor extending along the neurovascular bundle and in continuity with the median nerve seemed to originate from the median nerve. After resection of the fragile fleshy mass, the marked swollen median nerve was also noticed (Figure 3). The median nerve was completely encased by the mass.

**Figure 4:**
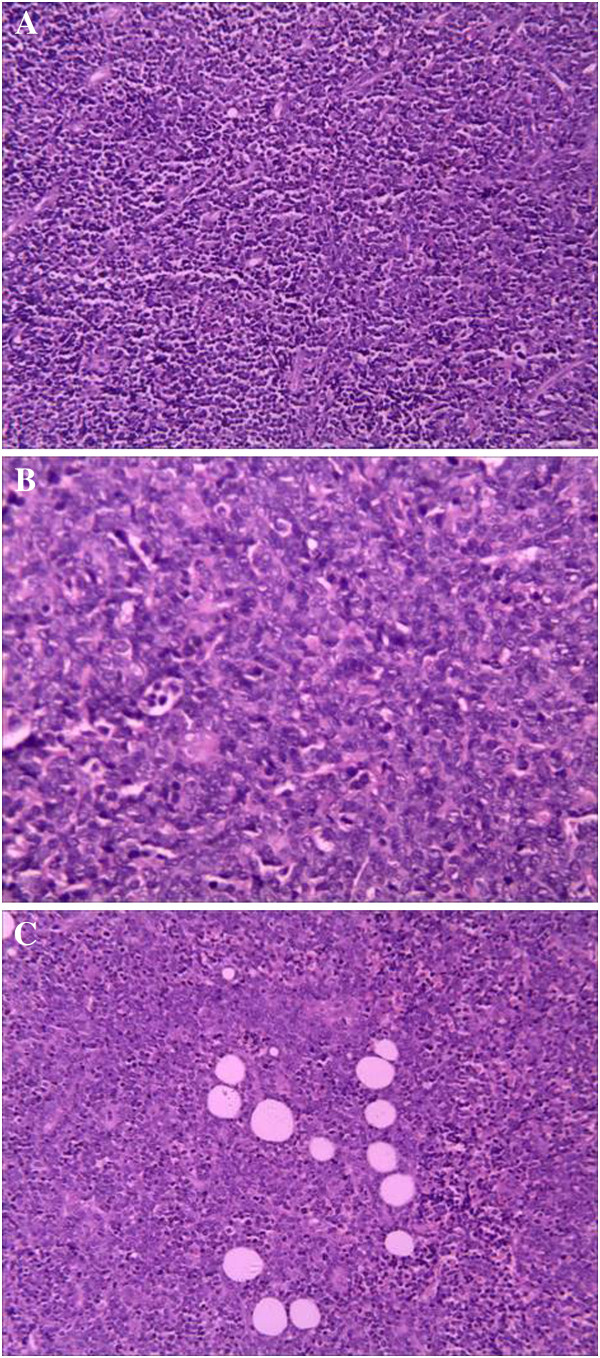
**Histologic analysis revealed monomorphic infiltration from lymphoblasts, showing scant cytoplasm and irregular nuclei.** (**A**) Diffuse, uniform, small-to-intermediated sized cells (H & E, ×200). (**B**) Scant cytoplasm, condensed chromatin and inconspicuous nucleoli; mitotically active lymphoma cells (H & E, ×400). (**C**) Infiltration of the fibro-fatty tissue (H & E, ×200).

Histologic examination revealed a diffuse dense monomorphic infiltration from small to intermediate-sized lymphoblasts (Figure 4A) showing scant cytoplasm and irregular nuclei with a high mitotic rate (Figure 4B), infiltrating among the fibro-fatty tissue (Figure 4C). Based on the immunohistochemical analysis, most of the lymphocytes stained positively for CD20, TDT, CD45, CD99, CD34, CD43, and negatively for CD3. Immunostaining for Ki-67 showed positive immunostaining of more than 90% of the lymphocytes. A diagnosis of precursor B-cell lymphoblastic lymphoma (PBLL) was rendered.

Clinical symptoms including severe pain and numbness were relieved soon after the surgical procedure of decompression. Local radiotherapy was applied to his left arm. PET and Gallium scintigraphy were not performed at diagnosis under cost constraints. He received two cycles of cyclophosphamide, doxorubicin, vincristine, and prednisone (CHOP) chemotherapy in the following 2 months. The patient was readmitted to our hospital due to persistent headache for a week before initiation of further chemotherapy. Cerebrospinal fluid (CSF) examination showed glucose 2.90 mmol/L, increased protein at 0.60 g/L, 90% mononuclear cells, 10% polykaryocytes, karyocyte count 90 × 10^6^/L, and no red blood cells (RBCs). The diagnosis of central nervous system (CNS) involvement was established. MRI of the brain was unremarkable. Neck, chest, and abdomino-pelvic sonography revealed no other involvement site except for a few axillary lymph nodes less than 10 mm in size. The patient died of multiple organ failure early in the course of high-dose methylprednisolone treatment shortly after being readmitted. Request for autopsy was denied.

## Discussion

LBL is a rare disease that shares the same biologic features as the acute lymphoblastic leukemia (ALL). Indeed, LBL and ALL are considered to be a single entity (lymphoblastic leukemia/lymphoma T and B types) in the World Health Organization (WHO) classification of precursor lymphoid neoplasms [4]. LBL is committed to the T-cell lineage (T-LBL), B-(B-LBL), or NK-cell lineage (NK-LBL) with an incidence of less than 2% within all NHLs, 1.7% for T- and less than 1% for B-lymphoblastic lymphoma respectively, even fewer for NK-lymphoblastic lymphoma [2]. T-cell type usually presents with a mediastinal mass and cervical lymphadenopathy. However, B-LBL often occurs within skin and bone involvement, except for a small proportion involving soft tissue and presenting with a mass lesion [5,6]. Localized invasion of peripheral nerves of extra-nodal LBL, a unique uncommon subtype, was termed as neurolymphomatosis [7].

The presenting signs and symptoms with LBL are shown in the primary sites where the disease is involved [8]. However, the clinical signs and symptoms are usually unspecific, which may pose a diagnostic problem. A technologically less complex needle biopsy may yield a diagnosis. However, our case was not possible to safely perform this procedure, as the biopsy may place the patient at very high risk of nerve and vessel injury. Therefore, a more invasive procedure should be performed for more reliable diagnosis. Imaging studies play a very important role to illustrate the relationship between the lesion and the adjacent tissue before the operation.

The MRI findings in our patient are similar to those of musculoskeletal lymphoma in the previous studies [9,10], such as predominantly homogeneous, intermediate signal intensity between those of fat and muscle on the T2-weighted image, and equal signal intensity compared with normal muscle on the T1-weighted image. The uncommon growth pattern of extension along the neurovascular bundle may represent infiltration along the lymphatic system.

The advantage of sonography in comparison with MRI is its ability to trace the imaged nerve segments in a single study and with superior resolution [11]. The encasement of the median nerve with loss of fascicular discrimination resembled the sonography of peripheral nerve compression, which seemed to occur secondary to schwannoma deriving from the nerve sheath [12]. However, three differences from nerve compression were still considered in this case. First, the sonographic appearance of the transverse median nerve was round-shaped in our patient, which was different from the somewhat flattened appearance in the entrapment neuropathies. Second, the nerve commonly showed a focal thinning in diameter at the site of direct compression, while our patient demonstrated marked swelling of the median nerve without reduction in nerve diameter. Third, color Doppler sonography in our patient revealed scarce intra- and perineural Doppler signals in the median nerve, whereas an increase in intraneural and perineural flow signals in color Doppler or power Doppler images was commonly seen in nerve compression [11].

PBLL is a highly aggressive subtype of lymphoma, with frequent bone marrow involvement, and a 5% to 10% incidence of CNS involvement [1]. Positive CNS involvement is defined as the presence of 5 × 10^6^/L or more white blood cells (WBCs) on CSF cytospin. The presence of CNS leukemia is an adverse prognostic factor and a potentially fatal complication of lymphoma [13]. The main cause of death in neurolymphomatosis is disseminated disease with associated multi-organ failure [14], as was seen in our patient. Peripheral neuropathy as a complication of lymphoma is not common, affecting only 0.1% to 2.0% of patients. The tumor may arise within aberrant lymph nodes, behaving as an infiltrative lesion instead of a compartmental condition [4,5]. The variable cells might possess stronger affinity to endothelial cells at the blood-nerve barrier, which would allow easier passage of the lymphoma cells into the peripheral nervous system. Sonographic and MRI assessment of the extent of the damaged median nerve in our patient was very beneficial. Explorative surgical treatment was very challenging in this case. Nonetheless the reasons for the procedure may be discerned in general: Firstly, proven median nerve compression inside the lesion surgical decompression was advocated; Secondly, an exact diagnostic work-up was mandatory to achieve an establishment of reliably histopathologic diagnosis.

## Conclusions

We experienced a PBLL presenting as a mass, and posing diagnostic difficulties for the referring clinician and radiologist. This could be mistaken for an enlarged neurogenic tumor.

## Consent

Written informed consent was obtained from the patient’s parents for publication of this case report and any accompanying images. A copy of the written consent is available for review by the Editor-in-Chief of this journal.

## Abbreviations

ALL, Acute lymphoblastic leukemia; CNS, Central nervous system; CSF, Cerebrospinal fluid; H & E, Haematoxylin and eosin; LBL, Lymphoblastic lymphoma; LDH, Serum lactate dehydrogenase; MRI, Magnetic resonance imaging; NHL, Non-Hodgkin’s lymphoma; PBLL, Precursor B-cell lymphoblastic lymphoma; RBC, Red blood cell; US, Ultrasound; WBC, White blood cells; WHO, World Health Organization.

## Competing interests

The authors declare that they have no competing interests.

## Authors’ contributions

XFS carried out the study design and writing. WYL and WG helped to draft the manuscript and participated in the literature search. XY performed the operation on the patient. FX performed acquisition of data and helped to draft the manuscript. All authors read and approved the final manuscript.
